# Supplementary material for: Tead1a Initiates Transcriptional Priming Through the TEAD1a/YAP‐Notch1‐Spi1/Cebpα Axis to Promote Neutrophil Fate
Source: Adv Sci (Weinh). 2025 Aug 28;12(41):e05441. doi: 10.1002/advs.202505441 (PMC12591127; doi:10.1002/advs.202505441)
Supplement: Supplementary file 4 — Supplementary Dataset [file ADVS-12-e05441-s004.zip › 3-4.zip!/3-4/02-4.cluster/cluster10/Enrich/ego_MF.pdf]

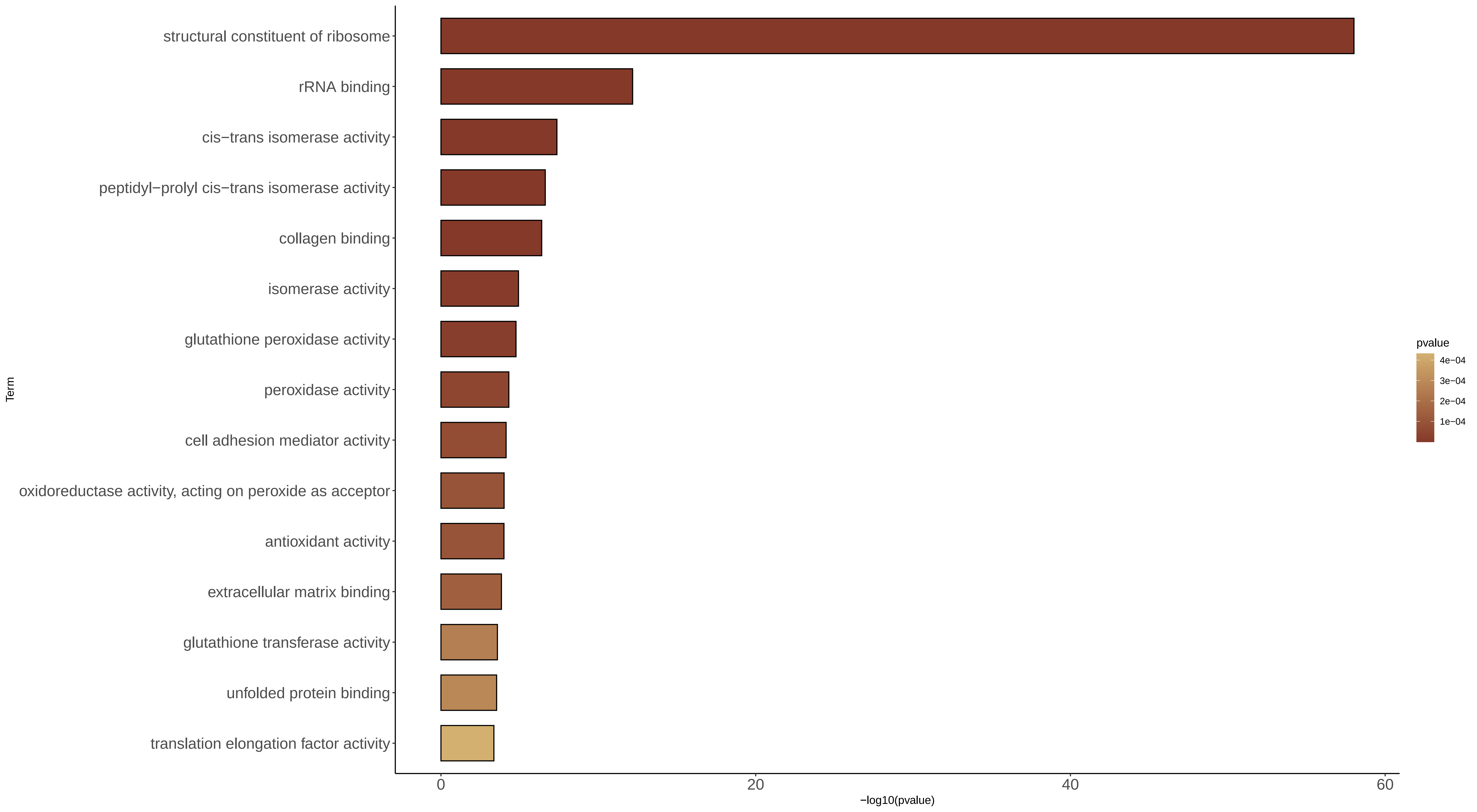
structural constituent of ribosome
rRNA binding
cis−trans isomerase activity
peptidyl−prolyl cis−trans isomerase activity
collagen binding
isomerase activity
glutathione peroxidase activity
peroxidase activity
cell adhesion mediator activity
oxidoreductase activity, acting on peroxide as acceptor
antioxidant activity
extracellular matrix binding
glutathione transferase activity
unfolded protein binding
translation elongation factor activity
Term
0
20
40
60
−log10(pvalue)
pvalue
4e−04
3e−04
2e−04
1e−04
